# Central lymph node metastasis is predictive of survival in advanced gastric cancer patients treated with D2 lymphadenectomy

**DOI:** 10.1186/s12876-020-01578-4

**Published:** 2021-01-06

**Authors:** Huiwen Lu, Bochao Zhao, Rui Huang, Yimeng Sun, Zirui Zhu, Huimian Xu, Baojun Huang

**Affiliations:** 1 Department of Surgical Oncology, First Affiliated Hospital of China Medical University, No. 155 Nanjing North Street, Heping District, Shenyang, 110001 People’s Republic of China; 2 Department of Clinical Medicine of Year 2017, Dalian Medical University, Dalian, People’s Republic of China

**Keywords:** Gastric cancer, Lymph node metastasis, D2 lymphadenectomy, Survival

## Abstract

**Background:**

The number of positive lymph nodes, which was defined as “N stage”, is mostly used to predict the survival of D2-resected gastric cancer patients, not the location. A “central lymph node” (CnLN) was defined by Ikoma et al., included common hepatic, celiac and proximal splenic artery LNs. CnLNs located in the extraperigastric area are included in the D2 LN station for gastric cancer. We speculate that CnLNs can be regarded as a predictor of survival.

**Methods:**

Eligible advanced gastric cancer patients who underwent curative resection and D2 lymph node dissection between 2004 and 2012 at our institution were identified. The frequency of CnLN metastases and risk factors affecting DFS were examined. Survival differences were assessed by log-rank tests and Kaplan–Meier curves.

**Results:**

The study identified 1178 patients who underwent curative surgery or D2 or more extensive lymphadenectomy. A total of 342 patients had been proven to have CnLN metastasis. Larger tumor size (*P* < 0.001), more frequent lymphatic vessel invasion (*P* < 0.001), signet ring cell histology (*P* = 0.014), and more advanced pathological T stage (*P* = 0.013) were significantly related to CnLNs metastasis. The patients with CnLN metastasis had a poor prognosis (HR for DFS of 1.366, 95%CI = 1.138–1.640, *P* = 0.001). For the pN2/3 patients, CnLN metastasis was associated with shorter 5-year DFS (for pN2 patients: 25.9% vs 39.3%, *P* = 0.017; for pN3 patients: 11.5% vs 23.4%, *P* = 0.005).

**Conclusion:**

Gastric cancer patients with CnLN metastasis who underwent D2 resection had a poor prognosis. With the same N stage, the patients with positive CnLNs had shorter survival. CnLNs metastasis could be a supplement to N stage and a predictor of survival in gastric cancer patients. Large sample, multicenter, randomized clinical trials are still needed in the future.

## Background

Despite a decline trend in its overall incidence, gastric cancer remains the second leading cause of cancer-related death worldwide according to the Global Cancer Statistics 2018 [1]. As a clinical doctor, our evaluation of survival outcome for advanced gastric cancer patients who were treated with curative surgery and lymphadenectomy is always dependent on accurate pathological tumor staging. TNM staging, which was originally published in 1966 and has undergone several revisions, is a widely applicable classification used to guide clinical practice [2]. The definitions of “T” and “M” stage have remained almost consistent, but the “N” stage has experienced a change from the “location” to the “number” of positive lymph nodes (LNs). Before the 4th edition of the American Joint Committee on Cancer (AJCC) classification system and the 2nd edition of the Japanese classification system, the location of positive LNs was used to define the “N” stage, which was different from that defined by the Union International against Cancer (UICC) [3, 4]. Nevertheless, some studies have suggested that the number of positive LNs reflects the tumor burden more accurately [5, 6]. To build a homogeneous, reproducible and accurate staging system, the UICC and AJCC reached complete agreement in their definitions of TNM and stage groupings. Although the N” stage is currently defined according to the number of positive nodes, the impact of the location of positive nodes on survival outcome in gastric cancer patients is notable, as reported by several Japanese trials [7, 8].

Gastric cancer patients with lymph node metastasis have a poor prognosis. The knowledge gap between Western and Eastern countries has made lymph node dissection debatable. Curative resection and D2 lymph node dissection for advanced gastric cancer patients is recommended, especially in Eastern countries [9]. To the best of our knowledge, a large number of positive LNs represents a high pathological N stage and a poor prognosis, but the impact of the locations of positive LNs on survival outcome is unclear. Recently, Ikoma et al. has revealed that common hepatic (station no. 8), celiac (station no. 9) and proximal splenic artery (station no. 11p) LN metastasis was a reliable predictor for survival outcome in gastric cancer patients who treated with neoadjuvant chemotherapy, and the results demonstrated that the pN2 and pN3 patients with positive CnLNs experienced shorter survival than those without CnLN involvement [8, 10]. However, no report has demonstrated the impact among GC patients who underwent D2 lymphadenectomy, without neoadjuvant chemotherapy. Whether the postoperative evaluation of the status of LN metastasis should not only count the number of positive LNs but also consider their location is worthy of consideration. The purpose of our study was to determine how anatomical location of the positive LNs influence the survival outcome of gastric cancer patients who have undergone curative surgery and D2 lymphadenectomy.

## Methods

### Study population

We retrospectively identified gastric cancer patients treated with curative surgery and D2 lymphadenectomy at the Department of Surgical Oncology, the First Affiliated Hospital of China Medical University between May 2004 and May 2012. The criteria for eligible patients were as follows: (1) all patients were histologically proven to be primary advanced gastric cancer via hematoxylin–eosin staining after operation; (2) the curative surgery and D2 or more extensive lymphadenectomy were performed. The patients who underwent palliative gastrectomy or with clinical and radiological evidence of peritoneal dissemination or distant metastasis were excluded from this study. (3) The patients with the history of other malignant tumors or with neoadjuvant chemotherapy or who were lost to follow-up or died within 1 month after surgery were excluded. According to the eligibility criterion above mentioned, a total of gastric cancer 1345 patients were included in this study. This study was approved by the Institutional Review Board of the Ethics Committee of China Medical University.

The study was approved by the Clinical Research Ethics Committee of the First Affiliated Hospital of China Medical University (approval No. 2016-114).

### Postoperative examination of lymph nodes

A surgeon examined the lymph nodes around each patient’s tumor site immediately after surgery, sorted them by their location (marked beginning at no. 1), and then sent them for a pathological examination. Patients were divided into two groups according to the presence of central lymph node metastasis. “Central lymph node” was defined by Ikoma et al. [8], which included common hepatic artery, celiac artery, and proximal splenic artery LNs (station nos. 8, 9, and 11p). The pathological stage was determined by the 8th edition of TNM staging system of American Joint Commission on Cancer (AJCC) [11].

### Statistical methods

Continuous variables between CnLN-positive and CnLN-negative group were compared using the Mann–Whitney test, and Fisher’s exact test or the chi-square test was used to compare the differences between categorical variables. Potential variables were verified by multivariate analysis using binary logistic regression. Disease-free survival (DFS) was calculated using the Kaplan–Meier method. DFS was defined as the date from curative surgery of the primary tumor to the date of first relapse or death from any cause. Univariate and multivariate Cox proportional hazards regression models were used to determine independent prognostic factors for gastric cancer patients, and the hazard ratio (HR) and its 95%CI were estimated. All statistical analyses were performed using SPSS 22.0 statistical software. The *P*-value less than 0.05 was considered to be statistically significant.

## Results

### Patient characteristics

This patient cohort consisted of 337 females (28.6%) and 841 males (71.4%), with average age of 58.54 years (range 7–84). A total of 342 patients (29.0%, 342/1178) had been proven to have CnLN metastasis, and the incidence of central lymph node metastasis was relatively common in gastric cancer patients.

The average number of examined CnLNs was 2.31 (range 1–15). Among the patients with central lymph node metastasis, 258 patients had no. 8 LN metastasis (range 1–9), 100 patients had no. 9 LN metastasis (range 1–9) and 64 patients had no. 11 LN metastasis (range 1–12).

### Factors associated with CnLNs

Clinicopathologic characteristics of gastric cancer patients with and without central lymph node metastasis were summarized in the Table1. The results indicated that the CnLNs-positive patients had a larger primary tumor size (≤ 4 cm vs > 4 cm, 69.3% vs 55.1%, *P* < 0.001), larger number of positive lymph nodes (*P* < 0.001), higher proportion of undifferentiated type (67.5% vs 60.5%, *P* = 0.014) and signet ring cell histology (14.0% vs 8.5%, *P* = 0.004), more frequent lymphatic vessel invasion (37.4% vs 18.2%, *P* < 0.001) and more advanced pathological T stage (*P* < 0.001) as well as N stage (*P* < 0.001). However, the distribution of other clinicopathologic factors including age, sex, diagnosis year, the number of retrieved lymph nodes, tumor location and resection type were comparable between CnLN-positive and CnLN-negative patients. In a multivariate analysis, larger tumor size (*P* < 0.001), more frequent lymphatic vessel invasion (*P* < 0.001), signet ring cell histology (*P* = 0.014), and more advanced pathological T stage (*P* = 0.013) were the significant risk factors for CnLN metastasis (Table 2).

**Table 1:** Background characteristics of the patient, overall and by CnLN examination status (N = 1178)

Variable	CnLN examined			*P* value
	All patients (N = 1178)	Positive (N = 342)	Negative (N = 836)	
Age, average, years	58.54 (7–84)	58.39 (23–81)	58.60 (7–81)	0.258
< 60, N(%)	582 (49.4%)	169 (49.4%)	413 (49.4%)	0.997
≥ 60, N(%)	596 (50.6%)	173 (50.6%)	423 (50.6%)	
Gender, N (%)				
Female	337 (28.6%)	98 (28.7%)	239 (28.6%)	0.517
Male	841 (71.4%)	244 (71.3%)	597 (71.4%)	
Diagnosed year				
2004–2006	185 (15.7%)	61 (17.8%)	124 (14.8%)	0.245
2007–2009	359 (30.5%)	109 (31.9%)	250 (29.9%)	
2010–2012	634 (53.8%)	172 (50.3%)	462 (55.3%)	
Tumor size				
≤ 4 cm	480 (40.7%)	105 (30.7%)	375 (44.9%)	< 0.001
> 4 cm	698 (59.3%)	237 (69.3%)	461 (55.1%)	
Number of examined LN, average	22.74 (3–119)	25.70 (4–119)	21.53 (3–82)	0.174
Number of positive LN, average	6.01 (1–118)	12.20 (1–118)	3.47 (3–79)	< 0.001
Type of resection				
Total gastrectomy	202 (17.1%)	63 (18.4%)	139 (16.6%)	0.254
Subtotal gastrectomy	976 (82.9%)	279 (81.6%)	697 (83.4%)	
Location				
Antrum/pylorus	620 (52.6%)	183 (53.5%)	437 (52.3%)	0.428
Body/fundus	124 (10.5%)	32 (9.4%)	92 (11.0%)	
GEJ/cardia	152 (12.9%)	38 (11.1%)	114 (13.6%)	
Total	282 (23.9%)	89 (26.0%)	193 (23.1%)	
Histology grade				
Differentiated	441 (37.4%)	111 (32.5%)	330 (39.5%)	0.014
Un-differentiated	737 (62.6%)	231 (67.5%)	506 (60.5%)	
Signet ring cell				
Yes	119 (10.1%)	48 (14.0%)	71 (8.5%)	0.004
No	1059 (89.9%)	294 (86.0%)	765 (91.5%)	
Lymphatic vessel invasion				
Yes	280 (23.8%)	128 (37.4%)	152 (18.2%)	< 0.001
No	898 (76.2%)	214 (62.6%)	684 (81.8%)	
Pathological T stage				
T2	220 (18.7%)	41 (12.0%)	179 (21.4%)	< 0.001
T3	666 (56.5%)	194 (56.7%)	472 (56.5%)	
T4	292 (24.8%)	107 (31.3%)	185 (22.1%)	
Pathological N stage				
N0	323 (27.4%)	0 (0.0%)	323 (38.6%)	< 0.001
N1	219 (18.6%)	35 (10.2%)	184 (22.0%)	
N2	265 (22.5%)	84 (24.6%)	181 (21.7%)	
N3	371 (31.5%)	223 (65.2%)	148 (17.7%)

**Table 2:** Multivariate logistic analysis for CnLN metastasis

Variable	Odds ratio	95%CI	*P* value
Gender (female vs male)	1.077	0.800–1.449	0.625
Age (≥ 60 year vs < 60 year)	1.026	0.786–1.340	0.850
Tumor size (> 4 cm vs ≤ 4 cm)	1.689	1.266–2.254	< 0.001
Lymphatic vessel invasion (yes vs no)	2.558	1.911–3.425	< 0.001
Type of resection (total vs subtotal gastrectomy)	1.117	0.748–1.668	0.589
Location (ref. antrum/pylorus)			
Body/fundus	0.806	0.494–1.317	0.390
GEJ/cardia	0.649	0.421–1.000	0.050
Total	0.822	0.572–1.181	0.288
Histology type (un-differentiated vs differentiated)	1.093	0.815–1.466	0.551
Signet ring cell (yes vs no)	1.708	1.117–2.611	0.014
Pathological T stage (ref. T2)			
T3	1.565	1.051–2.330	0.027
T4	1.949	1.248–3.036	0.003

### Survival analysis

**Fig.1:**
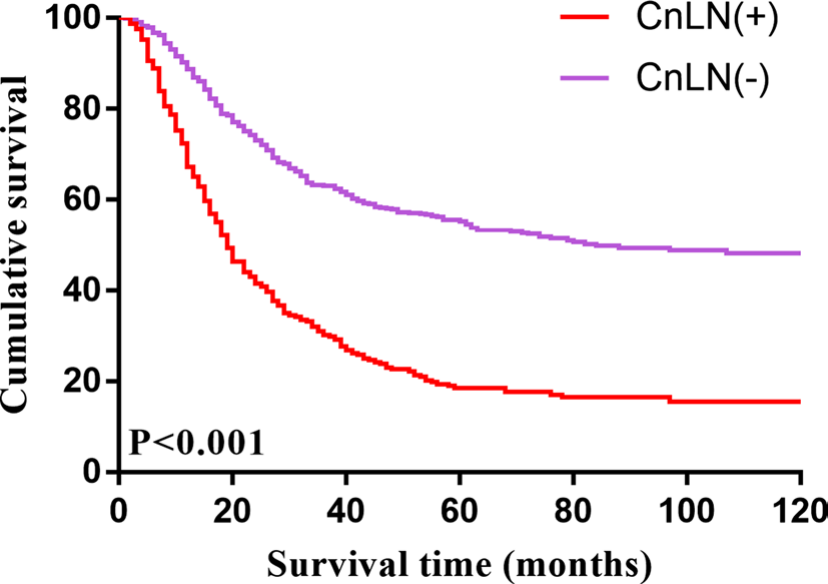
Kaplan–Meier estimate of overall survival by central lymph node positivity

**Fig.2:**
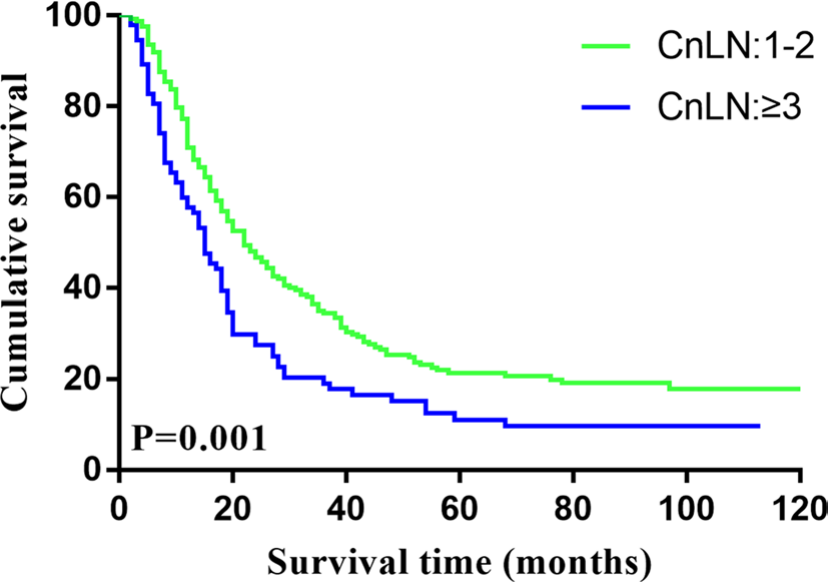
Kaplan–Meier estimate of overall survival by the number of positive central lymph node

**Fig.3:**
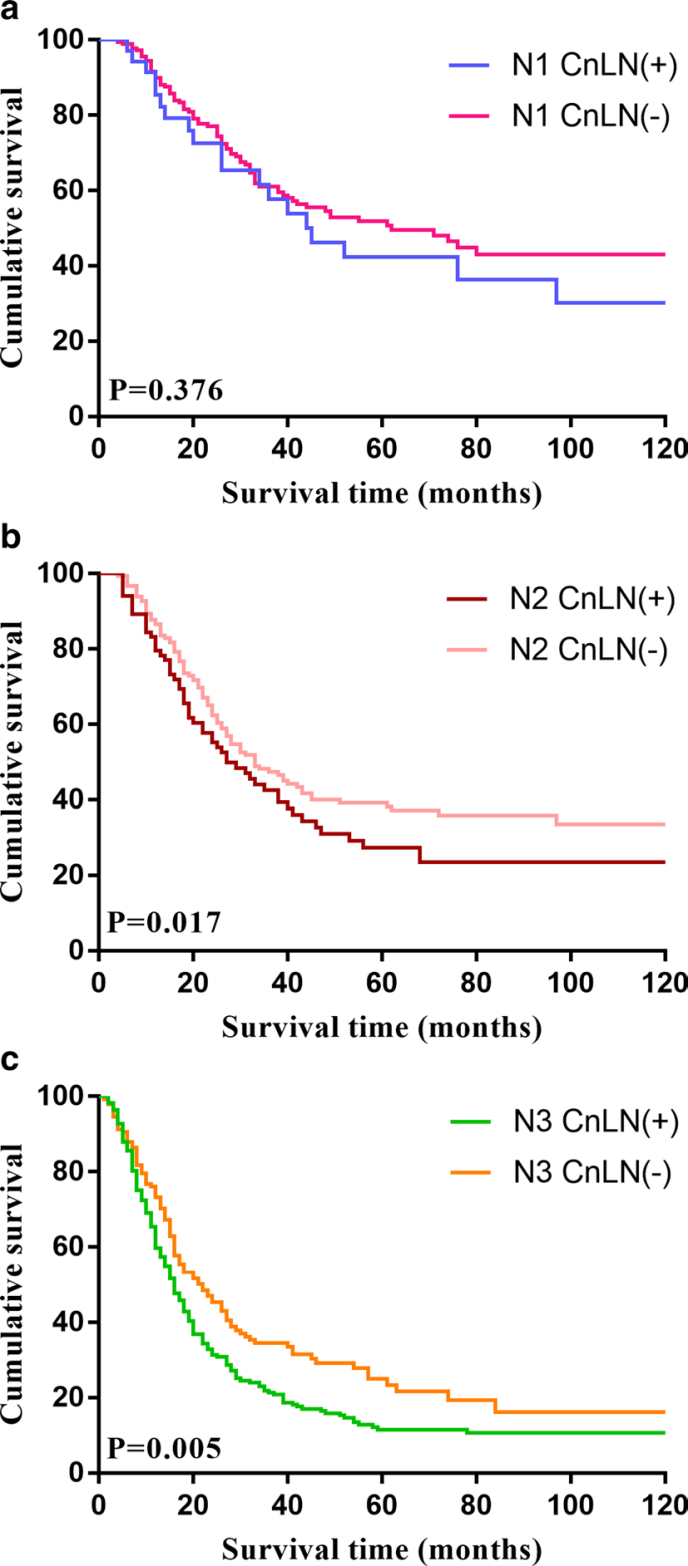
Kaplan–Meier estimate of overall survival by pathological N stage and central lymph node positivity

The average follow-up time was 39.91 months (range 1 to 178). The DFS rate for all 1178 enrolled patients was 82.5% at 1 year, 53.3% at 3 years, and 44.0% at 5 years. The CnLN-positive patients experienced a poorer survival than CnLN-negative patients (3-year DFS: 30.2% vs 63%; 5-year DFS: 18.5% vs 55.3%; *P* < 0.001). Kaplan–Meier curves for DFS are illustrated in Fig. 1. Moreover, the survival outcome of the patients with more than three positive CnLNs was worse than that of those with 1 or 2 positive CnLNs (3-year DFS: 19.0% vs 34.5%; 5-year DFS: 11.1% vs 21.4%, *P* = 0.001), Kaplan–Meier curves for DFS based on the numbers of positive CnLNs are shown in Fig. 2. In the subgroup analysis, the 5-year DFS rate of CnLN-positive and CnLN-negative patients with N1 stage was 42.3% and 50.6%, respectively; there was no significant survival difference between two patient groups(*P* = 0.376). For the N2 stage patients, the 5-year DFS rate of CnLN-positive and CnLN-negative patients was 25.9% and 39.3%, respectively (*P* = 0.017); the similar finding was observed in N3 patients (5-year DFS rate, CnLN-positive 11.5% vs CnLN-negative patients 23.4%, *P* = 0.005). Central lymph node metastasis had a significant prognostic significance for N2 and N3 stage patients. Kaplan–Meier curves for DFS based on the N stage are shown in Fig. 3.

Table 3 showed the results of Cox univariate and multivariate analysis for DFS. According to the univariate analysis, CnLN metastasis (*P* < 0.001), tumor size (*P* < 0.001), lymphatic vessel invasion (*P* < 0.001), resection type (*P* < 0.001), tumor location (*P* < 0.001), histology type (*P* < 0.001), signet ring cell histology (*P* < 0.001), pathological T stage (*P* < 0.001)and N stage (*P* < 0.001), and adjuvant chemotherapy (*P* = 0.049) were found to be significantly associated with poor survival outcome in gastric cancer patients. The multivariate analysis demonstrated that CnLN metastasis (HR:1.366, 95% CI 1.138–1.640, *P* < 0.001) as well as lymphatic vessel invasion (HR:1.402, 95% CI 1.168–1.683, *P* < 0.001), subtotal gastrectomy (HR:0.639, 95% CI 0.511–0.799, *P* < 0.001), undifferentiated type (HR 1.249, 95% CI 1.035–1.507, *P* = 0.021), signet ring cell histology (HR 1.206, 95% CI 1.001–1.452, *P* = 0.048), pathological T stage (T3 stage, HR:1.979, 95% CI 1.465–2.673, *P* < 0.001; T4 stage, HR:2.218, 95% CI 1.617–3.043, *P* < 0.001) and N stage (N1 stage, HR:2.858, 95% CI 2.047–3.990, *P* < 0.001; N2 stage, HR:3.373, 95% CI 2.451–4.640, *P* < 0.001; N3 stage, HR:5.469, 95% CI 3.967–7.541, *P* < 0.001), and adjuvant chemotherapy (HR 0.797, 95% CI 0.663–0.959, *P* = 0.016) were independent prognostic factors for DFS in gastric cancer patients.

**Table 3:** Univariate and multivariate Cox analysis of disease-free survival (DFS) in 1178 D2-resected gastric cancer patients

Variable	Univariate analysis		Multivariate analysis	
	HR (95%CI)	*P* value	HR (95%CI)	*P* value
CnLN metastasis (positive vs negative )	2.697 (2.290–3.176)	< 0.001	1.366 (1.138–1.640)	0.001
Age (≥ 60years vs < 60 years)	1.036 (0.882–1.217)	0.149	–	–
LN examined (≥ 15 vs <15)	1.246 (0.970–1.599)	0.085	–	–
Tumor size (> 4 cm vs ≤ 4cm)	1.592 (1.342–1.890)	< 0.001	0.991 (0.823–1.192)	0.922
Lymphatic vessel invasion (yes vs no)	2.295 (1.930–2.730)	< 0.001	1.402 (1.168–1.683)	< 0.001
Type of resection (total vs subtotal gastrectomy)	0.507 (0.419–0.613)	< 0.001	0.639 (0.511–0.799)	< 0.001
Location (ref. antrum/pylorus )				
Body/fundus	1.056 (0.801–1.393)	0.698	0.901 (0.668–1.215)	0.494
GEJ/cardia	1.381 (1.082–1.762)	0.009	1.353 (1.052–1.740)	0.019
Total	1.395 (1.151–1.692)	0.001	1.024 (0.819–1.279)	0.837
Histology type (un-differentiated vs differentiated )	1.499 (1.258–1.786)	< 0.001	1.206 (1.001–1.452)	0.048
Signet ring cell (yes vs no)	1.477 (1.170–1.865)	< 0.001	1.233 (0.962–1.580)	0.098
Pathological T stage (ref. T2)				
T3	2.647 (1.975–3.547)	< 0.001	1.979 (1.465–2.673)	< 0.001
T4	3.914 (2.888–5.304)	< 0.001	2.218 (1.617–3.043)	< 0.001
Pathological N stage (ref. N0)				
N1	3.066 (2.207–4.259)	< 0.001	2.858 (2.047–3.990)	< 0.001
N2	4.512 (3.320–6.132)	< 0.001	3.373 (2.451–4.640)	< 0.001
N3	8.403 (6.296–11.214)	< 0.001	5.469 (3.967–7.541)	< 0.001
Adjuvant chemotherapy (yes vs no)	0.834 (0.696–0.999)	0.049	0.797 (0.663–0.959)	0.016

## Discussion

R0 resection with D2 lymphadenectomy is significantly associated with improved survival outcome and widely used as a standard treatment for advanced gastric cancer patients in Eastern countries, especially China and Japan [9, 12, 13]. CnLNs (nos. 8, 9, and 11p) located in the extraperigastric area are included in the extent of D2 lymph node dissection for gastric cancer patients and routinely resected in clinical practice. Through pathologists’ hard work, tumors were staged correctly according to the TNM classification. To date, the definition of N stage was based on the number of positive lymph nodes. The later the N stage is, the poorer the prognosis. Few studies have focused on the location of positive lymph nodes and its impact on survival outcome in gastric cancer patients [5, 8]. However, whether the special locations of metastatic lymph nodes was associated with poor survival outcome of the patients treated with D2 lymphadenectomy remain controversial. In the present study, we found that CnLN metastasis was an independent prognostic factor for survival outcome in gastric cancer patients, especially when more than three positive CnLNs were observed (*P* = 0.001).

Ikoma et al. defined “central lymph node” as common hepatic artery, celiac artery, and proximal splenic artery LNs (station nos. 8, 9, and 11p) [8]. In our study, CnLN metastasis was relatively common (29.0%) (21.9% in no. 8 LNs, 8.4% in no. 9 LNs, and 5.4% in no. 11p LNs) and was significantly associated with poor survival outcome. And in this trial, larger tumor size (*P* < 0.001), more frequent lymphatic vessel invasion (*P* < 0.001), signet ring cell histology (*P* = 0.014), and more advanced pathological T stage (*P* = 0.013) were significantly related to CnLNs metastasis. In the subgroup analysis, we found that CnLN metastasis was associated with shorter 5-year DFS in pN2 and pN3 patients (for pN2 patients: 25.9% vs 39.3%, *P* = 0.017; for pN3: 11.5% vs 23.4%, *P* = 0.005), but not showed a significant difference for pN1 patients (42.3% vs 50.6%, *P* = 0.326). Central lymph node metastasis is predictive of prognosis for pN2/3 patients.

For pN1 patients, CnLN metastasis was not significantly associated with survival outcome, which might be related to the low metastatic rate and the mechanism of “skip metastasis” [14, 15]. Skip metastasis was defined when LN metastasis appeared to bypass or skip tiers rather than following the lymphatic streams and was not related to the location of the primary tumor. In earlier tumor stages, tumor cell colonization might be random, and studies have shown that station nos. 1, 7, 8a, 9, and 11 were the main sites of skip metastasis [16]. The pN1 stage patients with positive CnLNs were likely to experience skip metastasis. However, the association between skip metastasis and survival outcome in gastric cancer patients remains a matter of debate. Some studies have revealed that skip metastasis has no impact on survival [14, 16]. And our study supported this point, because CnLN metastasis in pN1 stage patients was not related to survival (*P* = 0.376). Skip metastasis might be the reason for which CnLN-positive pN1 patients did not experience poor survival rates, similar to pN2/3 patients.

According to the 5th Japanese gastric cancer treatment guideline, the National Comprehensive Cancer Network (NCCN), and the current American Joint Committee on Cancer (AJCC) staging system (8th edition), N stage is defined by the number, rather than the region, of positive lymph nodes among the regional lymph nodes, which is a consistent and effective method used worldwide [4, 11]. However, for patients with positive lymph nodes, the location of the metastatic LNs, especially central lymph nodes, is strongly correlated with survival [17, 18]. And in our study, we found the GC patients who located in the same N stage showed the different survival outcome because of CnLN metastasis. Especially for pN2 and pN3 stage, patients with positive CnLNs had shorter lifetime significantly. As a result, the N stage as well as central lymph nodes metastasis, both accommodate substantial importance when exploring the comprehensive treatment and predicting the prognosis for advanced gastric cancer patients. Additionally, central lymph nodes metastasis could serve as a potential supplement to the current international N stage for evaluating the prognosis of GC patients more accurately.

CnLN metastasis could be a potential predictor for prognosis and help guide postoperative treatment. Therefore, D2 lymphadenectomy and an accurate LN pathological examination are necessary for advanced gastric cancer patients [19]. Extensive lymphadenectomy could resect the micrometastasis and decrease the recurrence rate, especially for upper gastric cancer. Some studies have reported that upper gastric carcinoma is more prone to LN metastasis, especially at station nos. 1, 2, 3, and 7, and usually metastasizes to the para-aortic lymph node through the left gastric cancer artery and splenic artery [20, 21]. Thus, surgeons should carefully examine the lymph node and decide whether they need to perform a more extensive lymphadenectomy. In contrast to Eastern countries, D1 lymphadenectomy is more common in Western countries, mainly because of its lower rates of poorly differentiated histology and proximal stomach involvement—factors that are related to poor survival [6]. The standard range of lymphadenectomy is still under debate, but we suggest that patients with a later stage (i.e., later than T2) and with suspicious positive LNs should undergo extensive lymphadenectomy.

In clinical practice, gastric cancer patients, particularly those whose postoperative pathology reports have showed positive lymph nodes, tumor invaded the serosa and later TNM stage, are recommended to undergo adjuvant chemotherapy due to higher recurrent rate and poorer prognosis. Extensive and high-quality dissection and an accurate lymph node stage are key factors to consider when planning postoperative treatment. Guidelines request at least 15 lymph nodes to be examined in D2 resection for accurate LN staging [22]. And the later N stage, the more possible to metastasize to extraperigastric area following the lymphatic streams. In our study, we found that patients with more than three positive CnLNs had a poorer prognosis than the patients with one or two metastatic CnLNs (*P* = 0.001). Thus, in D2-resected GC patients in N2 stage or later, with positive CnLNs, especially at least three—all the variables related to a heavy lymph burden,—adjuvant chemoradiotherapy should initiate timely after surgery to eradicate micrometastasis and prolong survival. Some phase III studies have revealed that both adjuvant chemotherapy and radiochemotherapy were beneficial in preventing recurrence in D2-resected GC patients with positive LNs [23, 24]. The oxaliplatin combined with capecitabine (XELOX regimen) is commonly used as first-line postoperative adjuvant chemotherapy, especially for LN-positive gastric cancer patients, with tolerable side effects (most AEs are grade I/II) and a survival benefit (25.4–29 month) [25–27].

There were some limitations in our study. First, our study was a retrospective analysis involving a single institution. In the future, well-designed, large sample size and multicenter studies still need to be performed. Additionally, not all patients had at least 15 examined lymph nodes, which may have caused the incurrent N stage. Secondly, therapeutic protocols and recommendations for gastric cancer patients could have evolved during the study period. Adjuvant chemotherapy had been successfully performed in the recent decade, but the proportion of the patients who received adjuvant chemotherapy was relatively low in the current cohort. This may have a potential impact on prognostic assessment of gastric cancer patients.

## Conclusion

In conclusion, this study reported that CnLN metastasis could be regarded as a predictor for survival outcome in gastric cancer patients who underwent R0 resection and D2 lymphadenectomy. The anatomical location of positive LNs may be supplement to “N stage” for accurately prognostic evaluation. Large sample, multicenter, randomized clinical trials are still needed in the future.

## Data Availability

The datasets used and/or analysed during the current study are available from the corresponding author on reasonable request.

## Abbreviations

GC: Gastric cancer
DFS: Disease-free survival
OS: Overall survival
HR: Hazard ratio
LN: Lymph node
CnLN: Central lymph node
AJCC: American Joint Committee on Cancer
UICC: Union International Against Cancer
